# DTI-ALPS index decreased in patients with Type 2 Diabetes Mellitus

**DOI:** 10.3389/fnins.2024.1383780

**Published:** 2024-05-22

**Authors:** Shuncheng Yu, Hongjun Jiang, Langxuan Yu, Tieli Liu, Chun Yang, Jiajun Cao, Qingwei Song, Yanwei Miao, Weiwei Wang

**Affiliations:** ^1^Department of Radiology, The First Affiliated Hospital of Dalian Medical University, Dalian, Liaoning, China; ^2^College of Medical Imaging, Dalian Medical University, Dalian, Liaoning, China

**Keywords:** diffusion tensor image analysis of the perivascular space, glymphatic system, Type 2 Diabetes Mellitus, neurodegeneration, tract-based spatial statistics

## Abstract

**Backgrounds:**

Type 2 Diabetes Mellitus (T2DM) has become a significant global public health issue, characterized by a rising prevalence and associated deficits across multiple organ systems. Our study aims to utilize the DTI-ALPS technique to assess the change of ALPS index in T2DM patients, and to explore whether such changes are correlated with cognition level and diffusion parameters.

**Methods:**

The study involved 41 patients with T2DM (mean age, 60.49 ± 8.88 years) and 27 healthy controls (mean age, 58.00 ± 7.63 years). All subjects underwent MRI examination, cognitive assessment, and laboratory tests. Tract-based spatial statistics (TBSS) was used to evaluate white matter changes. GLM was performed to check the DTI-ALPS index difference between T2DM and HC groups. Spearman correlation analysis and partial correlation analysis were used to analyze the correlation between the DTI-ALPS index and diffusion properties & cognitive scores.

**Results:**

The results show that the ALPS index was lower in T2DM patients. MoCA score was significantly correlated with the ALPS index. Patients with T2DM had a significant increase in both mean diffusivity (MD) and radial diffusivity (RD) and decrease in fractional anisotropy (FA) compared to the HC group.

**Conclusion:**

The results suggest that the ALPS index is decreased in T2DM patients and associates with cognitive level.

## 1 Introduction

T2DM is a metabolic disorder characterized by insulin resistance and decreased insulin secretion, leading to abnormal glucose metabolism and related metabolic disorders. T2DM is a chronic and progressive disease that can lead to various complications, including kidney disease, retinopathy, heart disease, neuropathy, and cerebral vascular disease (Deshpande et al., 2008; Zaccardi et al., 2016). T2DM is becoming increasingly prevalent worldwide, with high morbidity and mortality rates. Epidemiologic studies have shown that individuals with diabetes have an increased risk developing dementia (Biessels et al., 2006). Studies have also shown that T2DM is associated with cognitive decline in multiple domains and structural abnormalities in the brain (Moheet et al., 2015). It has been suggested that the association between T2DM and dementia may be related to disorders of the cerebral glymphatic system (Pruzin et al., 2018). There have been many previous studies focused on the glymphatic system, which is important in transporting nutrients throughout the brain, and eliminating waste products generated by the central nervous system (Yankova et al., 2021). It has been shown that the glymphatic system is involved in the clearance of key proteins in neurodegeneration, and impaired glymphatic-glymphatic transport accelerates abnormal protein accumulation and cognitive decline in mouse models of Alzheimer’s disease, traumatic brain injury, and Parkinson’s disease (Iliff et al., 2014; Xu et al., 2015; Da Mesquita et al., 2018; Zou et al., 2019).

Researchers have used intrathecal contrast injections to visualize the clearance of the glymphatic system in the brain. However, this method is invasive and requires multiple intrathecal injections followed by MRI acquisitions (Iliff et al., 2012). Serious neurotoxic complications may occur when the dose exceeds a certain level (Patel et al., 2020). For the past few years, DTI-ALPS has emerged as a noninvasive technique for assessing the conditions related to the glymphatic system, providing an indirect index-the ALPS index, without the need for contrast injection (Taoka et al., 2017; Zhang et al., 2021). Many researchers have subsequently used this technique to study neurodegenerative diseases such as Alzheimer’s disease, Parkinson’s disease, and other dementia (Shen et al., 2022; Hsu et al., 2023; Wu et al., 2024; Zhang et al., 2024). In recent years, researchers have begun to apply ALPS technology to Type 2 Diabetes Mellitus patients, as well as its relationship with insulin resistance and disease course (Yang et al., 2020; Tuerxun et al., 2024). However, researches on the ALPS index in T2DM patients are far from enough, and it is unknown whether there is a correlation between cognitive level and the ALPS index in T2DM patients. Our study aimed to investigate the ALPS index using this noninvasive method and explore the association between ALPS index and cognitive level in T2DM patients.

## 2 Materials and methods

### 2.1 Participants

This study is approved by the Ethics Committee of the First Affiliated Hospital of Dalian Medical University (Ethical Application Ref: YJ-KY-FB-2020-08). 41 T2DM patients and 27 healthy controls were recruited. All subjects participated in the study voluntarily and signed informed consent.

The inclusion criteria of the T2DM group are as follows: (1) meet the clinical diagnostic criteria for type 2 diabetes recommended by the American Diabetes Association (ADA) in 2014 (American Diabetes Association, 2014); (2) Right-handedness; (3) No contraindications of MRI scanning, screened using MRI safety questionnaire, and the volunteers signed for confirmation; (4) Routine CT/MRI examination of the head showed no abnormality or only mild white matter osteoporosis. The first inclusion criteria of the healthy control (HC) group is physical fitness, and the rest are the same as 2 to 5 items of the T2DM group inclusion criteria.

Exclusion criteria of T2DM group: (1) National Institute of Health stroke scale Score ≥ 2; (2) Thyroid and parathyroid dysfunction, complicated with tumors, kidney dialysis and pregnancy; (3) Color blindness and color discrimination disorder: screening with color blindness test chart (5th edition); (4) There is a history of hazardous drinking; A history of drug or poison abuse or dependence; (5) History of chronic central nervous system diseases, such as Parkinson’s disease, dementia, epilepsy, etc.; (6) Serious medical emergencies, such as moderate to severe anemia, heart, lung, liver and kidney failure, severe electrolyte disorders; (7) Current or past history of major physical diseases (such as cerebral hemorrhage, massive cerebral infarction, intracranial infection, chemical or drug toxic encephalopathy, malignant tumors, etc.), autoimmune diseases, and other forms of endocrine diseases; (8) Individuals and first-degree (children’s parents, siblings), second-degree (uncles, aunts, grandparents) or third-degree (Cousins/Cousins) relatives have a history of mental illness; (9) Current or past History of mental illness (craniocerebral injury with unconsciousness for more than 5 min, craniocerebral surgery, schizophrenia, mania, depression, etc.), screened using the concise International Neuropsychiatric Interview (MINI); (10) History of craniocerebral surgery. Exclusion criteria of HC group: (1) the same as 1 to 10 items of T2DM group; (2) abnormal development of brain structure.

### 2.2 Collection of clinical data

The demographic variables and laboratory indicators of all subjects were recorded within 1 week before or after MRI scanning, including age, gender, Body mass index (BMI), education level (in terms of years of education), fasting plasma glucose (FG), glycated Hemoglobin A1c (HbA1c), fasting Insulin (INS), low-density lipoprotein (LDL), high-density lipoprotein (HDL), total cholesterol (TChol), triglycerides (TG), and Homocysteic Acid (Hcy).

### 2.3 Neuropsychological tests

The Mini-Mental State Examination (MMSE) and The Montreal Cognitive Assessment (MoCA) were used to assess the Cognitive status of all subjects. The Symbol Digit Modalities test (SDMT) was used to evaluate the two cognitive fields of memory and execution which were easily damaged in the early stage of T2DM. The tests were completed by an experienced neuropsychiatrist and all the subjects and the neuropsychiatrist were blinded to the study design.

### 2.4 MR parameters

MRI data of all subjects were collected using a 3.0 T MR scanner (Philips, Ingenia CX) with 32 channel orthogonal head coil, including axial FFE T1 sequence, axial FFE T2 sequence, axial TSE Flair sequence, sagittal 3D T1 TFE sequence and axial DTI sequence (64 directions) The imaging parameters of T1WI were: repetition time = 297 ms, echo time = 6.9 ms, flip angle = 75°, slice thickness = 3 mm, field of view = 230 × 183 cm^2^, matrix = 232 × 178; T2 fluid-attenuated inversion recovery (T2 FLAIR): repetition time = 11,000 ms, echo time = 120 ms, field of view = 230 × 184 cm^2^, matrix size = 240 × 143, inversion time = 2,800 ms, slice thickness = 3.0 mm with no gap between slices. Diffusion tensor imaging (DTI): repetition time = 6,000 ms, echo time = 92 ms, slice thickness = 2 mm, matrix size = 128 × 128, field of view = 256 × 256 cm^2^. T2WI: repetition time = 1,186 ms echo time = 13.81 ms, field of view = 230 × 183 cm^2^, matrix size = 256 × 164, slice thickness = 3 mm with no slice gap. 3D T1WI: repetition time = 6.6 ms, echo time = 3 ms, flip angle = 12°, slice thickness = 1 mm, field of view = 256 × 256 cm^2^, matrix = 256 × 240.

### 2.5 DTI preprocessing

FSL software was applied for DTI data preprocessing. Mricron was used to convert the original DICOM images into NifTI (.nii) for data compression. Eddy current correction was applied to correct for eddy current distortions and movement during acquisition. The resulting eddy correction output was then used to rotate the BVECs. Subsequently, BET brain extraction was performed with a b-value of 0 to obtain a mask of the brain, followed by tensor calculation (DTIFIT) to obtain the anisotropy index. The diffusion tensor of each voxel was fitted using the DTIFIT tool to generate fractional anisotropy (FA), mean diffusivity (MD), eigenvalues (λ1, λ2, λ3), Dxx, Dyy, and Dzz maps. Axial diffusion (Axial diffusion = λ1) and radial diffusion [RD = (λ2 + λ3)/2] coefficient maps were then calculated from these eigenvalues.

### 2.6 Tract-based spatial statistics analysis

The FMRIB58 FA standard space image was used as the target of TBSS, and all FA images were aligned to a standard space of 1 × 1 × 1mm. The average FA image and FA skeleton were created, and the FA value of each subject was registered to the average FA skeleton with a threshold of 0.2 for the FA skeleton. Next, a “distance map” was created from the skeleton mask. This was used for FA projection onto the skeleton. Finally, the script took a 4D all_FA image (containing the aligned FA data for all subjects), and, for each “time point” (i.e., subject ID), projected the FA data onto the average FA skeleton. This produced a 4D image file containing the skeleton of the (projected) FA data. The same FA transformation was then also applied to MD, Axial diffusion, and RD.

Age and gender were entered as covariates into the design matrix and contrast files before processing. Results are reported at the *p* < 0.05 level after 5,000 permutations using permutation-based non-parametric inference, with threshold-free cluster enhancement (TFCE) and family-wise error (FWE) rate correction for multiple comparisons. The FSL’s cluster was used to identify statistically significant (*p* < 0.05) clusters followed by an atlas query to describe the localization of all the anatomical clusters using the John Hopkins University (JHU)—white matter tractography atlas template.

### 2.7 Diffusion tensor image analysis along the perivascular space (DTI-ALPS)

**Figure 1:**
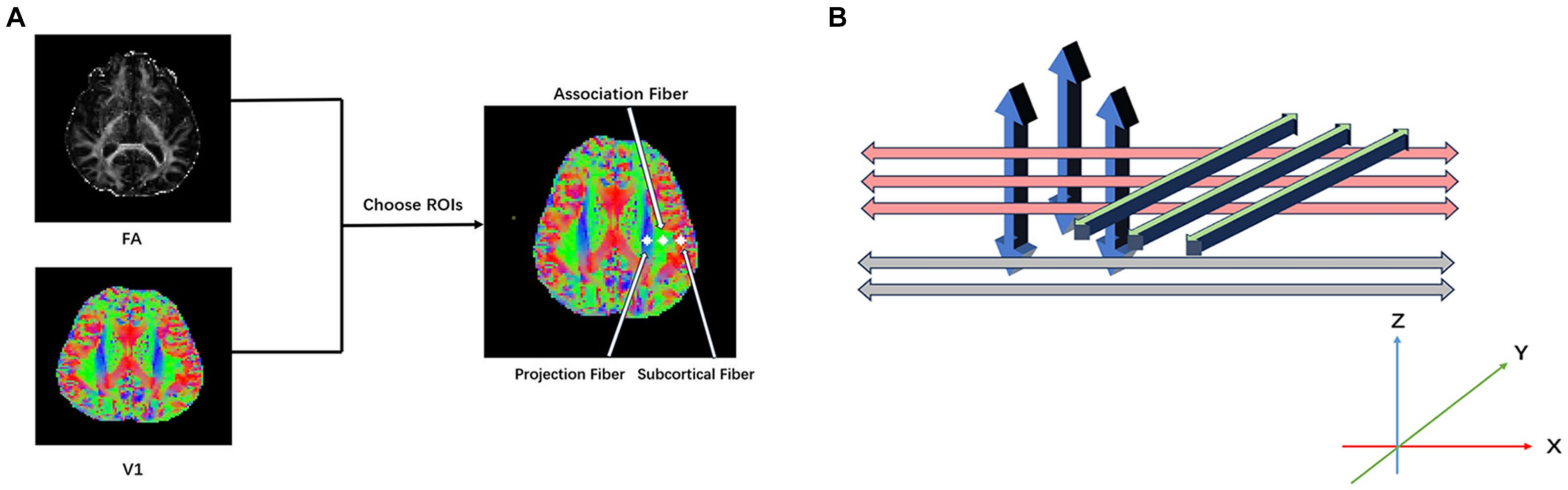
**(A)** Diffusion tensor imaging along the perivascular space (DTI-ALPS) index processing. Place the ROIs in the areas with projection fibers (blue), association fibers (green), and subcortical fibers (red) to obtain the fiber orientation and diffusivities along the *x*-, *y*-, and *z*-axes as voxel levels within the ROIs. **(B)** Illustrates the relationship between perivascular space (gray), projection fibers (blue), and association fibers (green).

The ROIs on projection and association fibers were mapped using the FSLeyes software (Figure 1A). Mappings of water molecule diffusivity along the *x*, *y*, and *z* directions of projection and association fibers were processed using DPABI. Circle ROIs of 6-mm diameters were drawn on the DTI fractional anisotropy (FA) map, and fiber orientations and diffusivities along the *x*-, *y*-, and *z*-axes were measured at voxel levels within the ROIs (Figure 1B). For each fiber on the same *x*-axis (projection, association, and subcortical fibers), one voxel was selected to show the maximum orientation. Dx values in the projection (Dxproj) and association (Dxassoc) neural fiber areas on the Dx map, Dy values in two fiber areas (Dyproj, Dyassoc) on the Dy map, and Dz values in two fiber areas (Dzproj, Dzassoc) on the Dz map were automatically measured to calculate the ALPS index using the following formula:

ALPS index = mean(Dxproj, Dxassoc)/mean(Dyproj, Dzassoc).

### 2.8 Statistical analysis

Statistical analysis was performed on SPSS (version 27, IBM Corporation, USA). The normality test of variables was performed using Shapiro–Wilk test. Independent two-sample t-test or Mann–Whitney U-tests were used to compare normally or non-normally distributed variables of the demographic characteristics of two groups. Chi-squared test was performed in the comparisons of categorical data. Independent two-sample t-test was used for the DTI-ALPS index comparison of the two groups. Then, GLM was performed to check the ALPS index difference between T2DM and HC groups. This approach controlled for various factors including age, gender, years of education, BMI, triglyceride, total cholesterol, low-density lipoprotein, and high-density lipoprotein. We conducted a test for multicollinearity among these covariates by checking the Variance Inflation Factor (VIF) for each covariate. The results indicated that all variables had VIF values less than 5, showing that multicollinearity did not significantly affect the integrity of our statistical model.

In the T2DM group, we applied spearman correlation analysis to analyze the correlation among ALPS index with TBSS diffusion parameters, and cognitive scores. Furthermore, partial correlation analysis was conducted to validate the correlation. For all statistical evaluations, a *p*-value <0.05 was deemed to indicate statistically significant.

## 3 Results

### 3.1 Participant characteristics

Sixty-eight subjects were collected in this study, including 41 T2DM patients (20 women and 21 men, age range 40 to 71 years, with a mean of 60.49 ± 8.88 years) and 27 age-, gender-, and education-matched healthy controls (17 women and 10 men, age range 40–67 years, with a mean of 58.00 ± 7.63 years). The two groups differed in BMI, HbA1c, FG, Triglyceride, and HDL, no difference were found in age, gender, years of education, insulin, Cholesterol, low-densitylipoprotein, MoCA, MMSE, and SDMT score (Table 1).

**Table 1:** The demographic, clinical, and laboratory data of the participants.

Characteristic	T2DM(*n* = 41)	HC(*n* = 27)	*p* value
Age	60.49 ± 8.88	58.00 ± 7.63	0.065^a^
Female gender, *N* (%)	20(48.78%)	17(62.96%)	0.251^b^
BMI	25.48 ± 3.09	23.60 ± 3.22	**0.018** ^**c**^
HbA1c	7.58 ± 1.45	5.50 ± 0.26	**<0.01** ^**a**^
Insulin	9.13 ± 8.67	6.78 ± 3.92	0.236^a^
FG	8.24 ± 2.58	4.88 ± 0.46	**<0.01** ^**a**^
Cholesterol	4.72 ± 0.95	5.01 ± 0.92	0.215^c^
Triglyceride	1.82 ± 1.28	1.22 ± 0.63	**0.013** ^**a**^
HDL	1.06 ± 0.25	1.29 ± 0.28	**<0.01** ^**c**^
LDL	2.57 ± 0.60	2.74 ± 0.65	0.281^c^
Hcy	12.32 ± 3.43	12.53 ± 3.17	0.806^c^
SDMT	33.97 ± 2.41	41.02 ± 2.69	0.06^c^
MOCA	25.42 ± 2.37	26.33 ± 2.57	0.073^a^
MMSE	28.49 ± 3.45	28.85 ± 0.91	0.054^a^

SDMT, Symbol Digit Modalities Test; MMSE, Mini-Mental State Examination; MOCA, The Montreal Cognitive Assessment. HbA1c, Hemoglobin-A1c. FG, fasting plasma glucose; HDL, High-density lipoprotein; LDL, Low-density lipoprotein; Hcy, Homocysteic Acid; BMI, body mass index.

^a^*p* values for differences between T2DM and HC obtained using Mann–Whitney U test; ^b^*p* values obtained using *χ*2 test between the two groups; ^c^*p* values for comparisons between T2DM and HC obtained two-sample *t*-test. Bold means *p* value less than 0.05.

Values are reported as mean ± SD and as frequency (percentage) for the categorical variables.

### 3.2 Tract-based spatial statistics results

**Figure 2:**
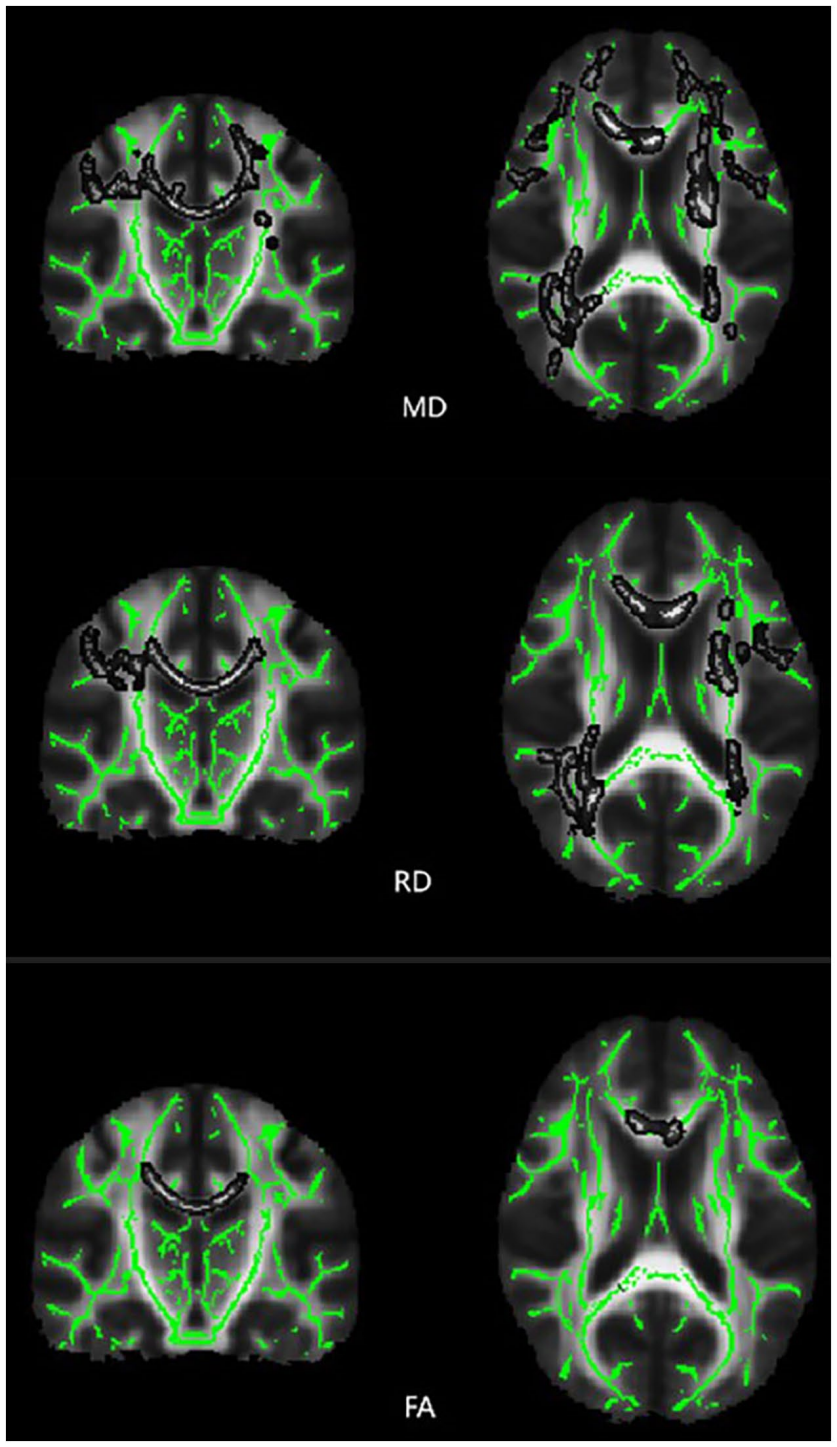
Differences in TBSS diffusion parameters values between the T2DM patients and HC group of subjects. Brain function color map, green area represents the mean FA skeleton, and gray area indicates regions with significant statistical values (*p* < 0.05, corrected by TFCE).

The results revealed significant differences in MD and FA between the T2DM and HC groups in one independent cluster, as well as two independent clusters with statistically significant RD values. Figure 2 and Table 2 show the detailed information between the HC and T2DM groups. MD and RD were increased in T2DM patients compared to the HC group, and FA was decreased in T2DM patients compared to the HC group (Table 2). There was no significant difference in axial diffusion.

**Table 2:** Cluster sizes and locations for voxels with significantly increased MD, RD, and significantly decreased FA between T2DM and HC groups.

Cluster	JHU WM tractography atlas	Voxel coordinates of Local maxima (MNI coordinates)			Voxel size	*p*-value
		*X*	*Y*	*Z*		
FA (cluster)	Anterior thalamic radiation R	94	125	98	2262	0.032
	Corticospinal tract L					
	Corticospinal tract R					
	Cingulum (cingulate gyrus) L					
	Cingulum (cingulate gyrus) R					
	Forceps minor					
	Superior longitudinal fasciculus R					
MD (cluster)	Anterior thalamic radiation L	88	114	97	19818	0.027
	Anterior thalamic radiation R					
	Corticospinal tract L					
	Corticospinal tract R					
	Cingulum (cingulate gyrus) L					
	Cingulum (cingulate gyrus) R					
	Cingulum (hippocampus) R					
	Forceps major					
	Forceps minor					
	Inferior fronto-occipital fasciculus L					
	Inferior fronto-occipital fasciculus R					
	Inferior longitudinal fasciculus L					
	Inferior longitudinal fasciculus R					
	Superior longitudinal fasciculus L					
	Superior longitudinal fasciculus R					
	Uncinate fasciculus L					
	Uncinate fasciculus R					
	Superior longitudinal fasciculus (temporal part) L					
	Superior longitudinal fasciculus (temporal part) R					
RD (cluster1)	Inferior fronto-occipital fasciculus L	130	75	104	234	0.048
	Inferior longitudinal fasciculus L					
	Superior longitudinal fasciculus L					
	Superior longitudinal fasciculus (temporal part) L					
RD (cluster2)	Anterior thalamic radiation L	91	112	97	12,168	0.026
	Anterior thalamic radiation R					
	Corticospinal tract L					
	Corticospinal tract R					
	Cingulum (cingulate gyrus) L					
	Cingulum (cingulate gyrus) R					
	Cingulum (hippocampus) R					
	Forceps major					
	Forceps minor					
	Inferior fronto-occipital fasciculus L					
	Anterior thalamic radiation L					
	Inferior fronto-occipital fasciculus R					
	Inferior longitudinal fasciculus L					
	Inferior longitudinal fasciculus R					
	Superior longitudinal fasciculus L					
	Superior longitudinal fasciculus R					
	Uncinate fasciculus L					
	Superior longitudinal fasciculus (temporal part) L					
	Superior longitudinal fasciculus (temporal part) R					

MNI, Montreal Neurological Institute; L, abbreviation for the left hemisphere, R, abbreviation for the right hemisphere; JHU-WM tractography atlas, John Hopkins University white matter tractography atlas; FA, fractional anisotropy, MD, mean diffusivity, RD, radial diffusion.

### 3.3 DTI-ALPS measurements

**Figure 3:**
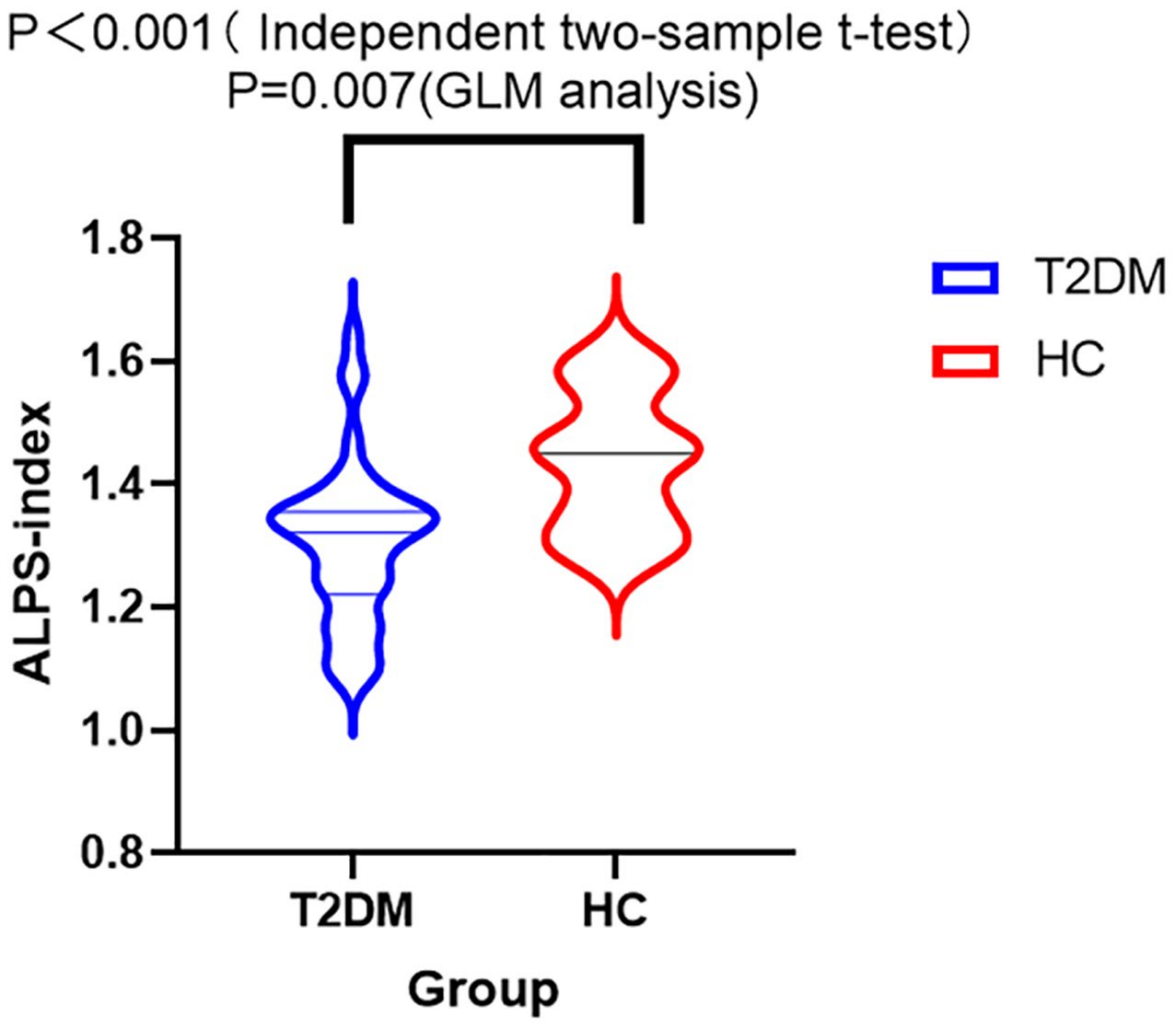
Violin diagram of the DTI-ALPS index between T2DM and HC. The *y*-axis displays the ALPS index, whereas the x-axis categorizes the groups. ALPS index was compared between T2DM and HC groups by GLM analysis with covariates including age, years of education, gender, BMI, total cholesterol, high-density lipoprotein, low-density lipoprotein, and triglyceride. There was a significant difference in the ALPS index across the two groups (*p* = 0.007). GLM analysis showed a lower ALPS index in the T2DM group than in the HC group.

Independent two-sample t-test revealed a lower ALPS index in the T2DM group compared to the HC group (*p* < 0.001). The DTI-ALPS values of the two groups are summarized in Figure 3. GLM analysis with covariates including age, gender, years of education, BMI, triglyceride, total cholesterol, low-density lipoprotein, and high-density lipoprotein revealed a lower ALPS index in the T2DM group compared to the HC group as well (*p* = 0.007) (Figure 3). The mean value of the ALPS index was still lower in T2DM (1.30 ± 0.13) than in HC (1.44 ± 0.11).

### 3.4 Correlation between the DTI-ALPS index and cognition

**Figure 4:**
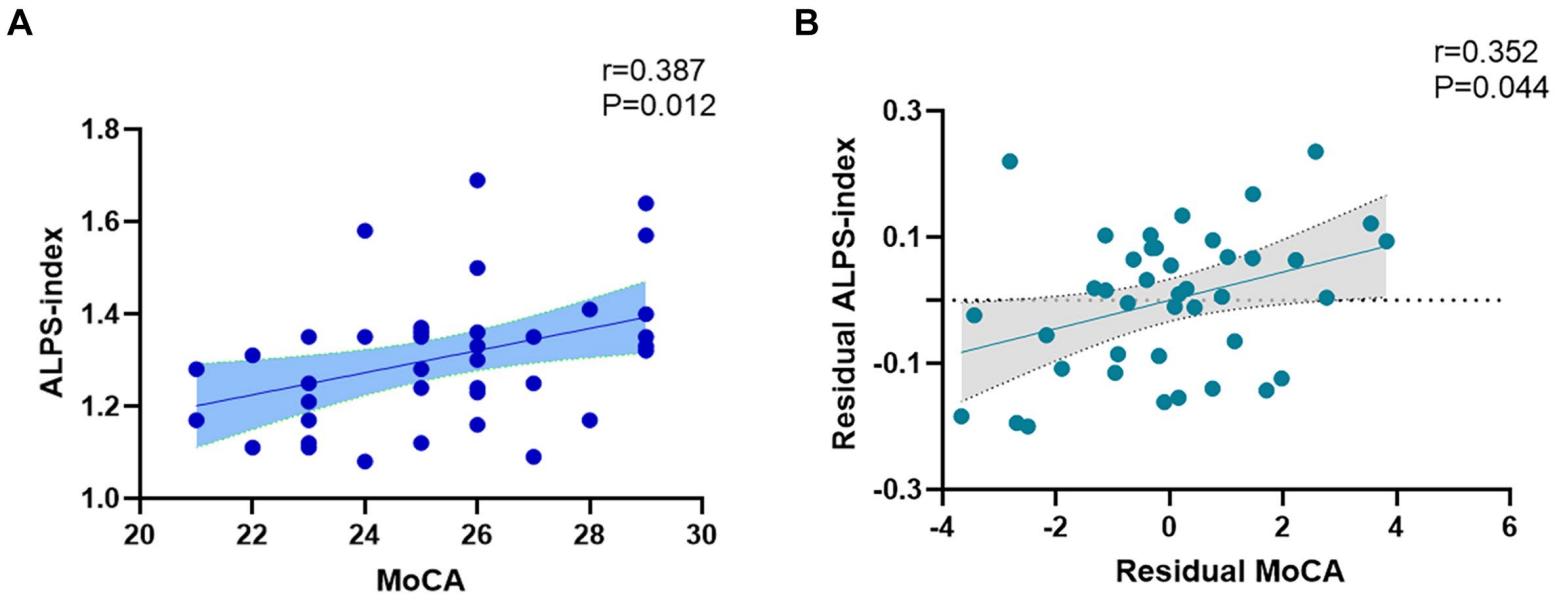
**(A)** Spearman correlation analysis revealed a positive correlation between ALPS index and MoCA score (*r* = 0.387, *p* = 0.012). **(B)** The association between MoCA and ALPS index. Residual MoCA and residual ALPS index represented MoCA and ALPS index corrected by age, gender, years of education, BMI, triglyceride, total cholesterol, low-density lipoprotein, and high-density lipoprotein (*r* = 0.352, *p* = 0.044).

The spearman analysis showed a positive correlation between ALPS index and MoCA score in the T2DM group (*r* = 0.387, *p* = 0.012) (Figure 4A). However, we did not find a correlation between ALPS index and MMSE score or SDMT score.

Partial correlation analysis showed that MoCA score still correlates with ALPS index after including age, gender, years of education, BMI, triglyceride, total cholesterol, low-density lipoprotein, and high-density lipoprotein as covariates (*r* = 0.352, *p* = 0.044) (Figure 4B).

### 3.5 Correlation between TBSS results and the DTI-ALPS index

Spearman correlation analysis showed that no correlation between the DTI-ALPS index and TBSS index were observed in T2DM group, including MD (*r* = 0.070, *p* = 0.666), RD cluster 1(*r* = 0.004, *p* = 0.981), RD cluster 2(*r* = 0.028, *p* = 0.860), FA (*r* = 0.012, *p* = 0.943). Partial correlation analysis showed that the ALPS index remained uncorrelated with the TBSS index, including MD (*r* = −0.124, *p* = 0.493), RD cluster 1 (*r* = −0.335, *p* = 0.057), RD cluster 2 (*r* = −0.195, *p* = 0.277), FA (*r* = 0.239, *p* = 0.180). Age, gender, years of education, BMI, triglyceride, total cholesterol, low-density lipoprotein, and high-density lipoprotein were set as covariates.

## 4 Discussion

In this study, we compared the DTI-ALPS index between T2DM patients and healthy controls, and investigated its association with cognitive scores in T2DM patients. First, we found that the mean value of the ALPS index in T2DM patients was lower compared to healthy controls. The ALPS technique has been used previously by researchers in neurodegenerative diseases such as Alzheimer’s disease and Parkinson’s disease. Other articles mentioned that the ALPS index was lower and significantly correlated with clinical motor and cognitive scores in Parkinson’s patients, suggesting a correlation between the degree of decrease in ALPS index and the severity of the disease (Bae et al., 2023). At this point, it is important to note the association between Alzheimer’s disease and T2DM. A search of literature revealed that Alzheimer’s disease and T2DM share some common features, such as chronic oxidative stress, impaired insulin signaling, and neuroinflammation, all of which are involved in disease progression (Chen et al., 2019). This point can be informative for the results obtained by DTI-ALPS in patients with T2DM. Besides, Yang et al. (2020) also used the DTI-ALPS technique to evaluate the ALPS index in the T2DM patients, and although different grouping methods were used in comparison, the results showed that the ALPS index of T2DM patients was lower than that of the HC. In an article published just last month (Tuerxun et al., 2024), the authors similarly analyzed the ALPS index in the T2DM patients by the DTI-ALPS technique, with the same result of a significantly lower ALPS index in T2DM patients. These findings are consistent with our result.

In 2017, Taoka proposed the concept of DTI-ALPS (Taoka et al., 2017) and in the following years, researchers applied the ALPS method to study kinds of diseases and believed that ALPS can indirectly reflect the function of the glymphatic system in the brain (Yang et al., 2020; Shen et al., 2022; Hsu et al., 2023; Tuerxun et al., 2024; Wu et al., 2024; Zhang et al., 2024). Regarding the impairment of the glymphatic system in T2DM, it has been demonstrated in animal modeling tests that more tracer is present in the paravascular vicinity and persistently retained in the hippocampus in rats with simulated type 2 diabetes compared to nondiabetic rats, and the quantitatively analyzed to show that there is a higher concentration of the residual agent and a slower clearance rate with simulated type 2 diabetic rats proves that T2DM has a detrimental impact on the functioning of the glymphatic system (Jiang et al., 2017). In our study, we indeed found a lower ALPS index in T2DM patients which seems to confirm the impairment of the glymphatic system in T2DM. Nevertheless, more and more controversies have been raised about DTI-ALPS method (Agarwal et al., 2024). Taoka believes that it’s improbable that the assessment using the ALPS technique, which is confined to a specific location, truly represents the operation of the brain’s entire glymphatic system. Research has corroborated that this waste clearance network performs a multitude of functions across different areas. Clearly, the phenomena detectable through ALPS represent just a fraction of these diverse activities (Taoka et al., 2024). The DTI-ALPS index probably cannot differentiate between perivascular water diffusion and other forms of directed water movement, like diffusion alongside fiber pathways, which are also included in the region of interest. While numerous investigations have indicated a correlation between this index and neurodegenerative disorders, it’s important to note that correlation does not imply causation, as the influence of confounding variables cannot be ruled out (Ringstad, 2024). The link between the ALPS index and human glymphatic system function has not yet been conclusively or rigorously confirmed through physiopathological research. Consequently, it is advised to treat the connection between the ALPS index and glymphatic drainage with a degree of caution (Wright et al., 2023; Haller et al., 2024). Therefore, we did not use a lower ALPS index to represent dysfunction of the glymphatic system. The pathological cause of the decrease of ALPS index needs further study. Pathophysiological studies about the relationship between ALPS index and glymphatic function are also needed in the future.

Second, we found a significant correlation between ALPS index and MoCA score in T2DM patients. Rapid population aging is currently a global phenomenon, and cognitive decline is becoming increasingly common (Livingston et al., 2020). Cognitive dysfunction is emerging as a critical comorbidity of T2DM. Studies have established that T2DM is linked to cognitive impairment (Koekkoek et al., 2015). T2DM accelerated the progression of hippocampus-associated cognitive decline in a middle-aged rat model of T2DM, which is equivalent to mimicking the deterioration of cognitive dysfunction in elderly T2DM patients (Zhang et al., 2016). Previous studies have shown that a decrease in the ALPS index is associated with cognitive decline in a variety of neurodegenerative diseases such as Alzheimer’s disease, PD, and idiopathic normal pressure hydrocephalus (Reeves et al., 2020; Chen et al., 2021). In patients with Alzheimer’s disease, it has been demonstrated that the ALPS index is significantly negatively correlated with amyloid and tau burden (Hsu et al., 2023). There are also researchers who have elucidated that the glymphatic system contributes to amyloid clearance in the mouse brain (Iliff et al., 2012). The glymphatic function of the central nervous system is an important component in the removal of metabolic wastes, and pathological proteins from the brain, and if there is a glymphatic dysfunction, it may lead to the deposition of metabolic wastes and pathological proteins, and then it can lead to cognitive impairment. Although patients with T2DM were not grouped according to cognitive level in our study, we found a significant correlation between ALPS index and MoCA score in T2DM patients. Our findings provide a possible indicator for future studies of cognitive function in T2DM using the DTI-ALPS technique.

Since studies have explored the relationship between whole-brain FA, MD, Axial Diffusion, and RD and composite scores for memory and executive function in Alzheimer’s disease patients (Mayo et al., 2019), which showed significant correlations between FA, MD, Axial Diffusion, and RD and executive functioning. Other scholars explored the correlation of ALPS index with FA, MD, Axial Diffusion, and RD in newly diagnosed focal epilepsy, the results showed a significant correlation, suggesting a correlation between the ALPS index and these parameters (Lee et al., 2022). So we want to explore the association between ALPS index and TBSS index. Our findings suggest that the correlation analysis between ALPS index and TBSS values showed no significant correlation between them. Because TBSS values can be used to reflect the degree of white matter damage (Smith et al., 2006), we thus hypothesized that in T2DM patients, white matter damage reflected by TBSS may not be related to decrease in ALPS index (Zhang et al., 2016).

Our study has several limitations. First, the sample size of this study was small and the ROIs were manually placed, which has a subjective element of measurement. Second, T2DM patients in our study did not show significant cognitive impairment based on cognitive scores, so we did not group them according to cognitive level, and thus did not observe differences in the ALPS index between T2DM patients with different cognitive levels.

## Data availability statement

The raw data supporting the conclusions of this article will be made available by the authors, without undue reservation.

## Ethics statement

The studies involving humans were approved by the Ethics Committee of the First Affiliated Hospital of Dalian Medical University (Ethical Application Ref: YJ-KY-FB-2020-08). The studies were conducted in accordance with the local legislation and institutional requirements. The participants provided their written informed consent to participate in this study. Written informed consent was obtained from the individual(s) for the publication of any potentially identifiable images or data included in this article.

## Author contributions

SY: Data curation, Writing – original draft. HJ: Writing – original draft. LY: Writing – original draft. TL: Data curation, Writing – original draft. CY: Data curation, Writing – original draft. JC: Resources, Writing – original draft. QS: Resources, Writing – original draft. YM: Supervision, Writing – review & editing. WW: Writing – review & editing.
